# The provision of dementia care in general practice: practice nurse perceptions of their role

**DOI:** 10.1186/s12875-021-01467-z

**Published:** 2021-06-09

**Authors:** Caroline Gibson, Dianne Goeman, Alison Hutchinson, Mark Yates, Dimity Pond

**Affiliations:** 1 Faculty of Health and Medicine, School of Medicine and Public Health, University of Newcastle, Melbourne, Australia; 2 Faculty of Health and Medicine, School of Medicine and Public Health, Central Clinical School, University of Newcastle, Monash University, Melbourne, Australia; 3 School of Nursing and Midwifery, Monash HealthCentre for Quality and Patient Safety ResearchInstitute for Health Transformation, Deakin University, Melbourne, Australia; 4 Deakin University School of Medicine, Ballarat Health Services, Melbourne, Australia

**Keywords:** Dementia care, People living with dementia, Primary care nurses, General practice

## Abstract

**Background:**

Primary care nurses can assist General Practitioner’s to identify cognition concerns and support patient health self-management for those experiencing cognitive impairment or dementia. This support may lead to more appropriate care and better health outcomes for this group. Consequently, there is a need to identify the role of the primary care nurse in dementia care provision, nurse perceptions of this role and to also understand the barriers and enablers that may influence any current or potential primary care nurse role in dementia care provision.

**Methods:**

Eight focus groups were conducted with a total of 36 primary care nurses. Data was transcribed verbatim and thematically analysed.

**Results:**

There was a high level of agreement between primary care nurses that they had a role in provision of dementia care. This role was largely attributed to the strong therapeutic relationship between nurses and patients. However, dementia care provision was not without its challenges, including a perceived lack of knowledge, limited resources and the hierarchical nature of general practice. Three main themes were identified: personal attributes of the primary care nurse; professional attributes of the primary care nurse role and the context of practice. Six sub-themes were identified: knowing the person; overcoming stigma; providing holistic care; knowing what to do; team culture and working in the system.

**Conclusions:**

The findings of this study suggest primary care nurses have a role in dementia care provision and, there is a need to provide support for the nurse to deliver person-centred health care in the context of cognitive impairment. As the demand for good quality primary care for people living with dementia increases, the role of the primary care nurse should be considered in primary care policy discussions. The knowledge gained from this study could be useful in informing dementia training content, to provide better prompts in the health assessment and care planning templates used by primary care nurses to better identify the care needs of people with a cognitive impairment and to develop dementia care guidelines for primary care nurses.

## Background

Due to the increasing economic and social impacts associated with aging populations, there is increasing health policy pressure, worldwide, to shift dementia care away from specialist secondary care into the primary care setting. [1, 2]. The benefits of a primary care led approach to dementia care include the potential provision of more holistic care [3] and a more cost-effective use of health care resources [4, 5]. However, a recent review of international literature revealed that primary care often fails people living with dementia [6–9]. Currently, both overseas and in Australia, dementia is reluctantly disclosed, poorly recognised, under diagnosed and less than optimally managed in the primary care setting [10].

While it is acknowledged that the management of dementia with its progressive cognitive, functional, physical and psychiatric changes is complex [10], people living with dementia (PLWD) have a fundamental human right to accessible, equitable primary care [11]. Barriers to the provision of optimal care include high care demands, requiring increased time, and continuing care which may result in the needs of people living with dementia not able to be met by the General Practitioner (GP) alone [1, 8, 12].

Early identification of cognitive changes and individualised care plans should reflect an understanding of how the person’s cognition may be influencing their self-care and adherence to health management strategies [13]. Whole-person dementia care that includes medical, social and psychological domains [14] and resists fragmented approaches to care [9] could lead to improved health outcomes for people with dementia and their family carers.

World-wide, nurses constitute the largest workforce in the primary care setting [15]. A recent systematic review investigating the roles of registered nurses in primary care across six countries found primary care nurses (PCNs) are responsible for clinical care, risk assessment, patient education and chronic disease management [16]. They also play a vital role in coordinating patient care before, during, and after the GP encounter [17] and nursing care has been identified as critical in meeting the health care needs and promoting quality of care for people living with dementia [18, 19].

The potential value of expanding the existing PCN role to include recognition and management of dementia has been acknowledged in international literature [12, 20, 21]. As co-morbidity in people living with dementia is high [22, 23], the PCN is likely to have established a therapeutic relationship with people with cognitive decline through routine primary care treatment, health assessments and chronic disease management. However, despite evidence supporting the involvement of the PCN in the recognition and care of people living with dementia and their support person(s), there is little evidence on the primary care nurse role in dementia care provision [24].

PCNs are an established workforce within primary care and accepted by patients, the community, General Practitioners and other health providers. Therefore, PCNs supporting the GP to identify cognition concerns and support patient health self-management in the context of cognitive impairment will likely lead to more appropriate care and better health outcomes for patients with existing or emerging cognitive impairment or dementia and their carers/family members. In 2015, Alzheimer’s Australia, now known as Dementia Australia, called for greater utilisation of nurses working in General Practice as a sustainable and cost-effective means of better meeting the health care needs for people with dementia and their support persons [25]. Mobilising this untapped PCN workforce has potential to reduce the time and cost needed to meet the health care needs of PLWD [1].

This paper reports on the perceptions of Australian primary care nurses, referred to as Practice Nurses (PN), and what they see as their role in the provision of dementia care in the General Practice setting and identification of barriers and enablers that may influence any potential role.

## Methods

### Research aim

In order to better understand PNs’ perceptions of their current and potential role in dementia care provision, the aims of this study were to:

1. identify the PN roles in dementia care provision
2. understand the barriers and enablers influencing the role of the PN in dementia care provision

### Design

A qualitative focus group methodology was used. Focus groups are facilitated group interviews that generate data through the opinions expressed by purposively selected participants individually and collectively on a particular topic of interest [26]. Focus groups are an efficient way to collect data [27] and are useful when current knowledge about a group or individual experience of a situation or event is limited because the group discussion can generate ideas that others can reflect on, and which some participants may not have explicitly thought about previously [28].

### Method Sampling

A purposive sample of PNs, qualified as a Registered or Enrolled Nurse, working in GP Clinics located in western Victoria, Australia, who attended a dementia education session prior to the focus group. Enrolled Nurses have completed a two year Diploma of Nursing and work under the supervision of a Registered Nurse.

### Recruitment of participants

Registered and Enrolled Nurses working in General Practice were invited to participate in the study, in conjunction with promotion of an education session, through the Western Victoria Primary Health Network social media and through direct invitations sent to individual GP Clinics by email and letter.

**Fig. 1:**
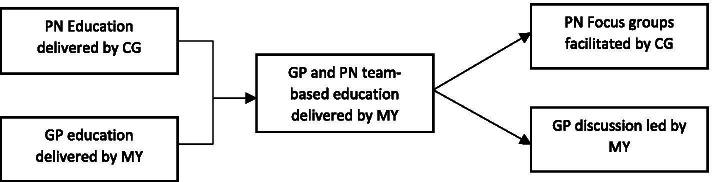
Illustrates the design of the education event with the inclusion of the focus group

Focus groups were conducted after each of eight education sessions titled “Cognitive Care in General Practice: Managing the whole patient pathway” scheduled at six sites in regional Victoria: Geelong, Ballarat, Horsham, Ararat, Daylesford and Warrnambool (Fig. 1). Two education sessions were conducted in each of Ballarat and Geelong and one session was conducted in each other location. The education sessions were conducted by two authors of this paper (CG, MY). Information on dementia and appropriate chronic disease management care planning for people for people with cognitive impairment in General Practice was provided. In Australia, GPs are funded under Medicare to develop chronic disease management care plan with eligible patients [29]. This is a documented plan of action developed collaboratively between the patient and the GP, or PN on behalf of the GP. The plan identifies individual health and care needs, describes individualised chronic disease self-management strategies, sets out the services to be provided by the GP and provides access to services that are required for ongoing maintenance of health issues.

The education sessions targeted both GPs and PNs and was supported by the Western Victorian Primary Health Network (WVPHN). The role of the Australian Commonwealth funded WVPHN is to facilitate the delivery of best practice primary health care across western Victoria which comprises 21 local government areas and a total population of approximately 618,000 people [30].

**Fig. 2:**
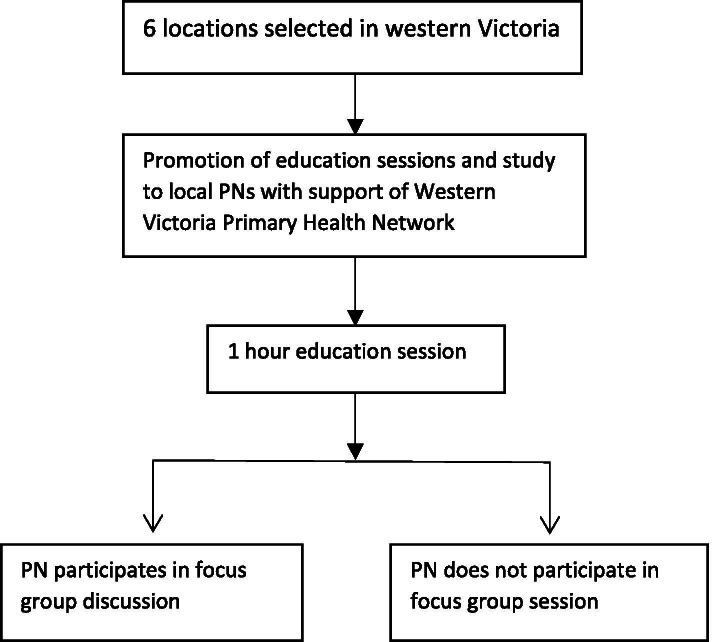
Illustrates the participant recruitment process

Nurses were given the option to leave prior to the commencement of the focus group. PNs who participated in the focus group discussion were offered a $25 Coles-Myer gift card in appreciation of their time to participate. This offer was included in the invitation to participate (Fig. 2).

### Data collection

The education and focus group events were held on mid-week evenings in September–October 2018. One hour was allowed for the education session and 45 min for the focus group with a supper break provided in between. The events were conducted in local venues including Western Victoria Primary Health Network meeting rooms and local community centres. In each setting participants sat around a table in clear view of each other.

The focus groups were moderated by a facilitator (CG, author and PhD student) and an assistant (LG, Registered Nurse with primary care experience) and followed a semi-structured interview guide with questions and prompts designed to elicit information on the role of the PN in dementia care provision. Both facilitator and assistant had completed a two-day group facilitation training workshop. All PNs who attended the education stayed for the focus group discussion.

A signed consent form and participant demographics sheet were collected prior to commencement of the focus group session. The focus group discussion was audio-recorded with consent and transcribed verbatim by a professional medical transcription service. Following each focus group, the facilitator and assistant shared observations of the focus group participant’s non‐verbal interactions and group dynamics. These observations were documented as field notes and supplemented the transcribed data. The transcripts were not checked by participants. As described in Sandelowski (1993) member validation is an on-going process and clarification of meaning was sought at the time of discussion [31].

### Interview guide

Six focus group questions were designed, by the authors, to probe participant experience on topics related to the research question. The questions (Table 1) were projected onto a screen in view of all participants to prompt discussion and maintain focus. Spontaneity of discussion was encouraged, and the facilitator actively drew participants back to the study topic only if the conversation was clearly irrelevant to the study topic or when there was repetition of the same issue.

**Table 1:** Focus group interview guide

● Do you discuss cognitive impairment with your patients?
● How would you describe dementia appropriate care/ cognitively aware chronic disease management?
● Is there value acknowledging dementia in care planning?
● Whose role is the recognition and management of dementia?
● What would be helpful in supporting you in dementia care provision?
● What prevents you providing dementia care?

### Focus group duration

The focus groups varied in duration from 23 – 42 min with an average of 33 min. (Total 260 min). Resource availability limited the number of focus groups to six, however data saturation had been reached at this time with similar discussion content emerging across all groups.

### Group dynamics

The contributions of participants were balanced across the focus groups. In one group there was a dominant participant who was focussed on one issue and provided little opportunity for others to contribute. This was moderated by re-iterating that all participant contributions were essential and shifting attention to other participants. In a different group a participant emailed the facilitator after the session to state she felt constrained in the conversation as her Practice Manager was present. This participant was invited to email any comments she would like to add to the facilitator, but no further information was forthcoming.

### Data analysis

Transcribed data were entered into the NVivo11 qualitative software [32] aiding data management and thematic analysis. Thematic coding of the focus group data followed Braun and Clarke’s [33] six phase process: familiarisation with the data, coding, generating initial themes, reviewing themes, defining and naming themes and writing up.

All authors read at least two transcripts and participated in an initial exploration of themes generated in a group discussion. Two coders (CG, AH) coded all the data, and a third coder (DG) was called upon to resolve any discrepancies in the coding. A detailed record of the data collection and data analysis phases was maintained to allow auditing of the data analysis process and findings. All data were available for the other study investigators to access.

### Trustworthiness

A rigorous approach to data analysis was achieved by systematically following Braun and Clarke’s method of thematic analysis [33]. This study meets Guba and Lincoln’s trustworthiness criteria: credibility, dependability, conformability, and transferability and authenticity [34]. Credibility was achieved with two coders (CG, AH) independently coding the data which was then cross-checked for consistency. Any discrepancy was discussed until agreement about themes were reached. A reflexive attitude was maintained throughout, with the focus group facilitator (CG) acknowledging her own subjectivity as a nurse with PN experience and the influence that this may have had on the study. The focus group facilitator and assistant exchanged observations of the group dynamic and discussion after each session and notes were recorded. Dependability was met through prolonged engagement, observation and clarifying meaning with participants. Conformability and transferability were met with thick description using narratives and maintaining an audit trail. The use of participant quotes expressing feelings and emotions associated with their experiences provides authenticity.

### Patient and public involvement

There was no patient or public involvement in this study.

## Results

### Participants

Eight focus groups were conducted with PNs (N = 36 in total), with group sizes ranging from two to nine participants. All PNs who attended the education session elected to stay and participate in the focus group. There were no non-participants present.

Table 2 presents participant demographics.

**Table 2:** Participant characteristics

Characteristic	N (%)
**Age range (years)**	
20–29	2 (6%)
30–39	4 (11%)
40–49	5 (14%)
50–59	17 (47%)
60–69	8 (22%)
**Gender**	
Male	1 (3%)
Female	35 (97%)
**Qualification**	
Registered Nurse	30 (83%)
Enrolled Nurse	4 (11%)
**Years of practice as PN**	
Less than 1	5 (15%)
1–2	1 (3%)
2–5	6 (18%)
5-10	6 (18%)
> 10	16 (45%)
**Chronic disease management part of role**	
Yes	31 (86%)
No	5 (14%)
**Self-reported completion of any type of dementia training in addition to the education provided prior to the focus group**	
Yes	24 (67%)
No	12 (33%)

### Thematic findings

**Fig. 3:**
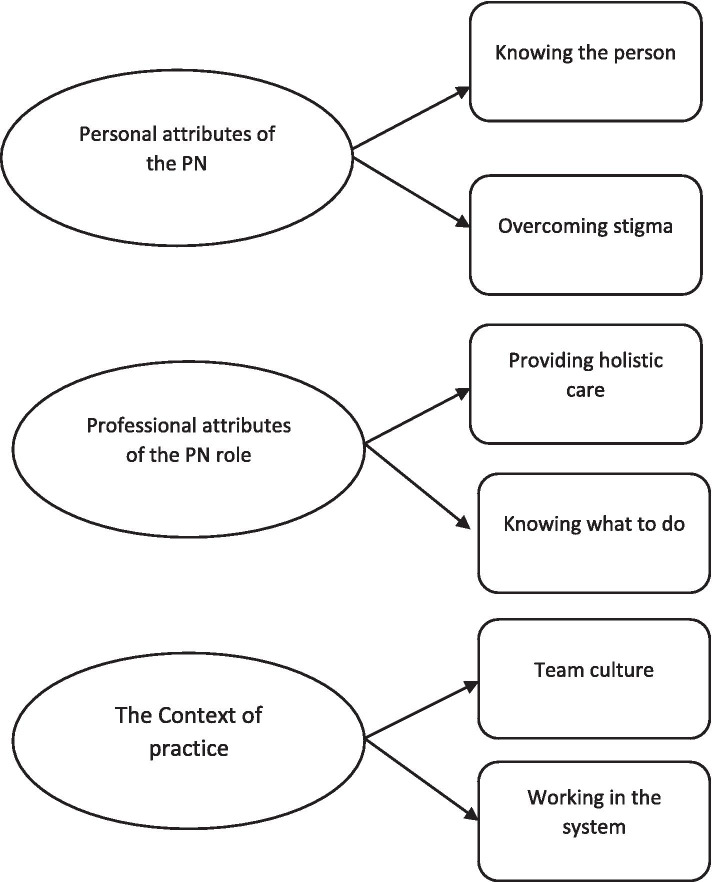
Final three themes with six sub-themes

Analysis of the data identified three themes with six sub-themes as illustrated in Fig. 3.

### Theme 1 Personal attributes of the PN

#### Knowing the person

There was a high level of agreement, across all FGs, that PNs have a therapeutic relationship with patients that supports the recognition and discussion of cognitive changes. The nurses described how knowing patients over a period of time provided the opportunity to notice changes in the patient’s presentation and behaviour.

“You’ve got this husband and wife coming in, and you know they’re falling apart because you see them all the time. She’s coming in all dishevelled.” (FG3)

“if they're doing something and then something changes, as in they used to be able to do it and then they couldn't do it … so I'm noticing this is going on today, how is everything? You don't seem yourself” (FG8)

All PNs described trust as a key characteristic of the nurse-patient relationship. This trust supported dementia care provision.

“there's a trust comes between you and the patient. They feel very threatened by families saying, mum, you're getting dementia. But you can - a nurse, the nurse can suggest little things and they don't feel as threatened” (FG6)

It was acknowledged in all FGs that both the GP and nurse have a role in the recognition of cognitive changes, but the nurse has the advantage having a different relationship, and also time, that supports disclosure.

*“But by gaining that therapeutic trust building up, that relationship with them. Having that extra time to listen to them ramble on a little bit. That's where we're probably going to get a better picture. Also having that little bit of extra time with them allows us visually to see how they behave, how they interact. How their thought processes work instead of just perhaps putting them on the spot in that brief GP type setting”(FG1)*

*“the GPs feel that their relationship can be threatened if they broach the subject …we have the gift of time … we can actually build rapport with the patient and speak to them”(FG2)*

#### Overcoming stigma

In all FGs, the nurses discussed the impact of negative assumptions associated with dementia in terms of both patient and nurse perceptions of dementia. Perceived stigma made conversation about dementia challenging, undermining provision of dementia care.

PNs were in high agreement that dementia care provision was limited when patients were reluctant to discuss cognitive changes as *“they still see it as a real fear”* (FG6) and *“one of the biggest barriers is patient acceptance”* (FG1). However, it was acknowledged that these difficult conversations needed to be had if appropriate care was to be provided.

*“if we’re all just having it* [the conversation with] *everybody… you know it becomes more normal…It’s a bit like depression; once upon a time, nobody talked about it. Well most people are happy to talk about it now that it’s not as scary. Whereas dementia is still pretty scary”(FG5)*

Some PNs, across all FGs, demonstrated their own negative attitudes to people living with dementia, potentially undermining good dementia care provision. The nurses generally used positive language but there were occasions when inappropriate language was used; for example, *“this patient's saying, no, I'm all fine. I'm thinking, you're as demented as anything”* (FG6). When asked about possible barriers to developing chronic disease management care plans with people living with dementia, it was suggested that a care plan could not be developed because the nurse may not be *“Getting a straight story”* (FG1) or concerns that *“What they're telling you is the truth, for one”* (FG7).

### Theme 2 Professional attributes of the PN role

#### Caring holistically

All focus group participants described their nursing care as holistic and that this approach supported the provision of dementia care.

*“when I first started nursing it was very task orientated. You told the patient what to do and they were expected to do it. But now the push is for the holistic view. So you’re looking at not just the disease, you’re looking at them as a person and their whole lifestyle. So even if you’re not thinking cognitively, if you are thinking of the holistic patient, the cognitive stuff starts popping up”(FG3)*

When explored more closely, although the PNs perceived their care as holistic, the majority of nurses described disease specific and clinically focussed care planning.

*“I haven't probably pushed enough in that cognitive sort of side of things. I'm really concentrating on the physical, you know, medical things”(FG8)*

Despite the disease focussed nature of care plans if the PN was to include cognition most nurses stated they would not address cognition separately.

*“All our care plans are based around musculoskeletal, cardiovascular or diabetes. I don't think I'd do it separately, I'd incorporate it into everything else”(FG7)*

One group did spend some time exploring whether a cognition-specific care plan would be useful.

*“it’s such a big topic it could be - it could be a care plan or whatever on its own… Is there an actual assessment you can do separately?”(FG2)*

The majority of PNs saw the assessment and management of the impact of cognitive impairment as an additional task rather than something to integrate throughout chronic disease education and management.

*“they are coming for generally a specific thing like diabetes or something like that. So, you do - you touch on their mood and that sort of stuff. Sometimes it can just be hard to move over to… When you’ve got so many other things you’ve got to look at”(FG2)*

By viewing cognitive assessment and management as an additional task, most nurses stated that it would add time that nurses did not have.

*“Sometimes time constraints would stop you. You haven’t got time to… sit with them and go through all the cognitive stuff”(FG4)*

Some nurses did not perceive time as a limitation, describing an opportunity to gather information about cognitive changes with subsequent visits potentially providing a more holistic approach.

*“We need a follow-up to see what changes have occurred and then we need to plan care, support and carer support appropriately”(FG2)*

Despite the limitations described by disease focussed care planning and perceived lack of time to address cognitive changes, the majority of PNs did see value in considering cognition in care planning as it was part of the person.

*“It doesn't matter where the cognition impairment comes, whether it's early in the cardiac disease, or late in the cardiac disease, we've got to…because it's combined as part of the same person, we've got to deal with it very early and make sure that we look after people early on(FG6)*

Exploring cognitive impairment in care planning was perceived to improve quality of care as it helped correctly identify the cause of health concerns leading to appropriate management.

*“I just think it’s a huge area that’s lacking and I think we can do a lot as a practice nurse…we’re seeing those patients coming with high blood pressure, high blood sugar, but why? Why are they missing the medication, why aren’t they - you know that sort of thing”(FG3)*

Despite PNs, across all FGs, describing their nursing practice as holistic, in only one focus group did nurses clearly articulate the need to address dementia in chronic disease management care planning. However, they did not describe how they might do this.

*“It isn't all just about facts and figures. It's not about the one disease or the one comorbidity, it's the whole lot impacting on each other … you can't isolate one disease from the other. You've got to - if someone's got dementia and diabetes, for example, you can't just treat the diabetes without having stuff in place for the dementia and likewise”(FG7)*

### Knowing what to do

There was a high level of agreement across all focus group participants that PNs needed to know more about recognising dementia.

*“I think we're recognising there is an increasing need of recognition because it's - a thing. It is a growing - it's increasing”(FG6)*

It was commonly acknowledged that asking about cognition was “as part of the whole package” (FG5) and that PNs “do not ask as regularly as we should” (FG8). The majority of PNs, however, admitted that they did not know what to do once cognitive impairment was identified.

*“I’m just wondering what, as a nurse… what can we do? Is it just referring on? Or is there stuff that we can do of value? … What is our role other than keeping an eye on them?(FG2)*

In many cases this lack of knowledge left them feeling powerless.

*“but I don’t know what to do with these people. The poor carer comes in and they’re nearly crying and pulling their hair out … but you still feel that you* [are] *useless because you don’t know what to do for them”(FG2)*

Lack of dementia knowledge and skills was often attributed to the perception that PNs are generalist health practitioners and that dementia care requires specialist knowledge.

*“we're jack of all trades and masters of none? Like you're not really focussing on one - you might go from that, to doing a four-year-old immunisation and wound care, to, you know”(FG6)*

A few PNs did not perceive the identification of a potential problem with memory as part of their role.

*“I don’t think that we should be in the role to say, yes I think you’ve got a problem with your memory”(FG2)*

And many PNs stated they would want a diagnosis listed in the medical record before they considered the impact of cognitive impairment when developing a care plan.

*“Yeah, when I do a care plan before I even get the patient in, I’ll sort of look at their problem list and I’ll sort of work out in my head some goals related to each problem that they’ve got. So then if it did have dementia there, I could think of something to do with dementia”(FG5)*

The majority of FG participants recognised that they needed to *“pick the right time to ask”* (FG1) about memory and this skill was often attributed to the nature of nursing and “*using that sort of sixth sense of nursing”* (FG8).

All PNs stated the 75 + Health Assessment, a general health assessment for people aged 75 years and older, was the most appropriate opportunity to discuss cognition. Nurses described it as better fit, when compared to chronic disease care planning, because there was a prompt to ask about cognitive change and do a cognition screening tool, the Mini-Mental-Scale-Examination.

*[Asking about cognition] “Particularly in the health assessments… I was going to say, not during a care plan. Yeah, definitely in a health assessment” because it’s listed on the template “You’ve sort of got that prompt to fill in”(FG4)*

All focus group participants agreed home visits to complete health assessments were optimal in recognising cognitive impairment.

The nurses also stated that changes could be picked up opportunistically in provision of clinical care.

*“you're doing a spirometry say and things weren't just making sense and it was really hard to get them to understand... you might be able to say, so, it seems like you're finding this a bit challenging. What's difficult here? You could probably start a conversation there that might lead you to have some suspicion that you may then raise with the GP”(FG8)*

When the PNs were prompted to consider how they would change care provision in response to identifying cognitive impairment, PNs in one focus group did not consider changes to usual care delivery and were largely in agreement that dementia care was primarily someone else’s responsibility, and saw their role as facilitating a referral to the Cognitive Dementia and Memory Service (CDAMS). CDAMS is a medical specialist diagnostic service in Victoria, Australia.

*“that’d be my first port of call would be actually to get a CDAMS assessment because … Have they got dementia or not … Yep, I think that’s the first port of call…When they’ve had that CDAMS assessment, come back and see me… When we get an outcome from that then there should be some recommendations or I would think from the CDAMS assessment... Yeah, well they [CDAMS] can refer or [I’m assuming] that they may have some case workers or something that they refer onto to make sure that sort of starts there…you can refer them onto a case worker that can get all those ducks in a row for them” (FG2)*

However, the majority of PNs did identify some specific things they would do in response to the recognition of cognitive impairment. These changes included modifying how they communicate with people if they identified cognitive impairment.

*“Well, I mean you would sort of…like your wording; make sure that its simple things I think you mostly would need to do; things that they would understand, yeah”(FG5)*

Many PNs across the FGs discussed how they would provide reassurance and support by making referrals.

*“the nurse reassuring the patient and the carer that there is help and we’ll refer you onto somebody that we feel can help you”(FG2)*

*“Getting them connected with services in the community, be it home help, Meals on Wheels, social groups”(FG7)*

The majority of PNs would support the patient in planning for the future. Planning for the future was described in terms of managing day-to-day activities such as shopping and medication administration. None of the participants referred to future health care directives or advance care planning.

*“planning really early and what plans have you got in place? How can we give them clues to deal with all of those things early on”(FG6)*

Determining patient supports when cognitive impairment was identified was another role many PNs took on.

*“finding out who their social supports are and working out - seeing if they’ll engage and assist with the care”(FG2)*

Most nurses recognised that supporting the carer was important.

*“you want to be able to maintain the carer, carer’s role and help support relationships there where you possibly can as well, because they’re - we all know what sort of stress these people are under and their families”(FG2)*

Education sessions were seen, by some PNs, as useful in increasing knowledge and skills; however all PNs stated that the use of templates with prompts would be most helpful in the provision of appropriate dementia care.

*“The prompting in the care plan itself, in the template. Like we have your height, your weight, let's put a cognitive assessment … Just to make you go, oh have I picked up anything while we've been discussing something…that doesn't quite make sense”(FG7)*

*“They can keep you focussed, a template, I reckon”(FG6)*

*“I think it’s a very sensitive subject… So, to actually have a list of questions that you could ask I think would be handy to - the language, the words, correct terminology, the words that you use - it’s not going to be threatening to them”(FG2)*

Having a specific dementia care guideline “*so we’re not messing it up”* (FG2) was also seen by most nurses as helpful in the provision of appropriate dementia care.

*“Just this sequencing of the progression of the condition, at what point do we … we need some sort of stage plan when we know when things get to this situation we need to refer onto so and so or we need to revisit that or where do we go to from here?”(FG2)*

### Theme 3 The context of practice

#### Team Culture

Working in practices with a strong team culture, in which the nurse felt respected and valued, supported collaborative care with the GP, potentially increasing the quality of care provided to PLWD.

*“I’ve worked with both extremes. You get the ones that are trusted and value their practice nurses…they read everything you write in the care plan and take it on board, to the other end of the spectrum…There’s still a few [GPs] around who think you’re a hand maiden…It’s how they value the team…Can you drag a GP across and go, I’m worried about Mr so and so, I’m a bit worried about his memory, its deteriorating. If the GP - if you’ve got a good team they’ll go, oh I’ll look into that the next time I see him” (FG3)*

Many of the nurse participants described working in medical practices where they were not supported to function as proactive health practitioners.

*“The thing is, we've got - we're limited to what we can do. We can suggest things, but we've sort of - you've got to bring it to the GP’s attention sort of thing if it's a patient. It's - we can't just go ahead and order things like that. It's got to come from the GP, so we're sort of…We can plant the seed, we plant the seed pretty much and say, you know, we're just a bit concerned about this patient such-and-such, or whatever, and then they – whatever”(FG6)*

Good communication and collaborative care with other health providers was valued in supporting the PN in dementia care provision.

*“It can be just people who see them regularly can be the first ones that pick up when things are wrong…I think you're right. The people that see them on a regular basis. The podiatrist … whereas I may never have seen them before, but they may have seen them over a period of time”(FG1)*

#### Working in the system

Working as a PN in small community practices was seen as supportive of good dementia care.

*“Us on this side of the table are in smaller communities and the communities, you are really aware, you know…you see Mrs such-and-such down the street, or you see her coming out of the pokies every day… you have a little bit more insight into what's actually going on…I've seen you driving up the wrong side of the road”(FG6)*

All FG nurses agreed that current primary care funding models, which do not financially remunerate most nursing activities, impact on nurse scope of practice and autonomy, potentially limiting their role in providing good dementia care.

*“Well from where we are at in a bush nursing centre, it’s obviously a little bit different to practice. Whereas we might see the patient more often than what you would in the medical centre. So we can actually see the changes differently, we talk to them a lot more, we go to their home a lot more. So we can certainly pick up a lot more issues and have triggers. We have a lot more time to spend with them. .. Yeah and a lot more time. We’re not financially [driven].We’re not looking at the dollar sign every five minutes as you would in the medical centre”(FG3)*

Perceived difficulty in getting a dementia diagnosis was seen as a barrier to the provision of dementia care. This was particularly significant given that many PNs wanted a diagnosis before they would consider cognitive impairment in the provision of care.

*“It's can be a bit of a quagmire, the process of getting them diagnosed … if you do have the concerns about their memory and then need them to go to the memory clinic… The waiting list ... the paperwork you have to fill in”(FG6)*

Given that many of the PNs in this study described their role in provision of dementia care as making referrals, the complexity of a health care system requiring lengthy referrals and multiple assessments was seen as a barrier to the provision of dementia care.

*“We get an hour, but half of that is if you have to write 10,000 referrals. It’s like I’ll go through My Aged Care, you need that extra time”(FG5)*

*“All the assessments they have to have, you know like to…It's just ridiculous. Just to even get a district nurse or…Early enough to be able to get support and medication… they've got to have a My Aged Care, or they're under 65 they've got to have this, Can't they take the word of a registered general nurse, who has assessed them as needing this care, but no, they've got to have three other assessments”(FG6)*

## Discussion

There was a high level of agreement between PNs that they had a role in provision of dementia care. Although varying in confidence, the PNs recognised opportunities in their usual nursing practice to identify cognitive impairment. The 75 + health assessment, particularly when conducted within the client home, was perceived as the optimal opportunity to identify cognitive impairment. In addition to identifying changes in memory, the nurses described referrals, guidance to community support services, future planning identifying patient supports, inclusion of the carer and simplifying language as the main features of their role in dementia care provision.

PNs routinely use health assessment and care planning templates, albeit disease focussed ones, and all nurse participants strongly agreed that templates with prompts would support the provision of dementia care and be easy to develop and implement on an individual practice level. The use of clear care pathways and/ or decision support tools have been shown to be effective in models of care for managing complex conditions[3] and could potentially be incorporated into routinely used care planning templates.

Many PNs wanted dementia care guidelines clearly describing “what to do at each stage of dementia”. Relying on guidelines to facilitate dementia care provision is potentially problematic, however. This is because guidelines have a variable impact on practice change [35] and may not support patient-centred care as they are necessarily reductionist in nature and do not take into account individual circumstances [36]. The PNs overwhelming described their care as holistic and person-centred, which along with the development of strong therapeutic relationships, was perceived to enable provision of dementia care. It is well established in the literature that therapeutic relationships and person-centred care contribute to holistic whole person care that is essential to dementia care [37–39]. However, rather than holistic and person-centred care, PNs in this study primarily described delivery of disease-focussed chronic disease management, which remains common practice in primary care [9]. This misalignment between nurses’ belief that their practice is person-centred and their actual practice was explored in a recent literature review which showed person-centred care in nursing is poorly defined and operationalised [40]. Few nurses in the present study suggested that a cognitive impairment would impact on all chronic disease management and thus should be integrated into all care provided. This is significant given that in primary care improving dementia care is best considered within a general approach across chronic conditions, since people living with dementia typically have multiple conditions to be managed [9, 41, 42]. No PN described how they would adapt chronic disease management in the context of cognitive impairment.

Consistent with the literature, the nurses in this study found asking about cognitive decline difficult, due to a lack of knowledge and experience [19], feelings of helplessness [43] and negative attitudes about people living with dementia which can lead to an unwillingness to get involved [14].

It is well known that high performing primary care encompasses a team-based approach [44]; however, findings of this study support evidence that many PNs and GPs, despite working in a multidisciplinary setting, do not provide collaborative care [45]. The PNs in this study who worked in settings where they felt respected and valued were more likely to communicate concerns about a person’s cognition to the GP and contribute suggestions for care potentially improving the quality of care provided to PLWD.

### Strengths and limitations

Focus group methods were used to facilitate primary care nurses to contribute, explore and clarify their views on a research topic that little is known about.

A strength of this study was the structured analysis process. The high level of agreement between the themes generated independently by the authors increase our confidence in the results.

The use of a convenience sample can decrease trustworthiness. However, including an experienced group of primary care nurses who could actively contribute to the discourse supported natural exchanges and exploration about their role in the care of people living with dementia.

When the group members know each other there is a risk that they may be less likely to be critical about their own and other’s practice. Other potential biases with focus groups include group think, the halo effect and the dominance effect [46]. The facilitator was observant to these possibilities and effective moderation ensured conversation was equally shared and robust in all groups. In an attempt to minimise power relations, only PNs were included; however, in one group a PN was also the Practice Manager. The facilitator was aware of this and provided one participant who may have been affected by this an alternative way to contribute to the discussion.

Holding the focus group after a dementia education session was a pragmatic approach to recruiting PNs. It is well known that primary care practitioners are difficult to recruit in research studies often because of pressures on clinician time. Combining the education session and the focus group presented an efficient use of time. It is acknowledged that the education material addressed prior to the focus group, and the group knowledge that the facilitator was a PN and PhD candidate may have influenced the ideas participants expressed in the focus group interviews and may have prompted socially desirable responses to questions. It is also possible that participants were more interested in the topic than the average primary care nurse.

### Implications

PNs have a role in dementia care provision and as the demand for good quality primary care for PLWD increases, the role of the PN should be considered in primary care policy discussions. The knowledge gained in this study could be used to review health assessment and care planning templates used by PNs to provide prompts to better identify the care needs of people with a cognitive impairment. Additionally, these findings may be useful in informing dementia training content and the development of dementia care guidelines for PNs.

## Conclusions

This study provides insight into the role of PNs in providing care to people living with dementia. The findings point to the need for further support for PNs to develop a role in dementia care provision and deliver person-centred health care in the context of cognitive impairment. The outcomes have significant potential to improve the care received by people living with dementia and the people who support them in General Practice.

## Data Availability

The transcripts analysed in this study are available from the corresponding author on reasonable request.

## Abbreviations

APNA: Australian Primary Health Care Nurse
CDAMS: Cognitive Dementia and Memory Service
GP: General Practitioner
PCN: Primary Care Nurse
PLWD: Person/ People living with Dementia
PN: Practice Nurse
WVPHN: Western Victoria Primary Health Network
